# Gender Differences in Mouth Opening on Temporomandibular Disorder Patients—Implications for Diagnosis

**DOI:** 10.3390/jcm14113865

**Published:** 2025-05-30

**Authors:** David Faustino Ângelo, Henrique José Cardoso, Ricardo São João, Carlos Brás-Geraldes, David Sanz, Francesco Maffia, Francisco Salvado

**Affiliations:** 1 Instituto Português da Face, 1500-493 Lisboa, Portugal; david.sanz@ipface.pt (D.S.); francesco.maffia@gmail.com (F.M.); 2 Centre for Rapid and Sustainable Product Development, Polytechnic Institute of Leiria, 2430-028 Marinha Grande, Portugal; 3 Faculty of Medicine of Lisboa University, 1649-028 Lisboa, Portugal; fjsalvado2002@yahoo.com; 4 Serviço de Estomatologia Hospital Egas Moniz—Centro Hospitalar de Lisboa Ocidental, 1349-019 Lisboa, Portugal; 5 Clinica Universitária de Estomatologia, Centro Hospitalar Universitário Lisboa Norte (CHUNL), 1349-019 Lisboa, Portugal; 6 Department of Computer Science and Quantitative Methods, School of Management and Technology, Polytechnic Institute of Santarém, 2001-904 Santarém, Portugal; ricardo.sjoao@esg.ipsantarem.pt; 7 CEAUL—Centro de Estatística e Aplicações, Faculdade de Ciências, Universidade de Lisboa, 1749-016 Lisboa, Portugal; carlos.geraldes@isel.pt; 8 Center for Global Studies (CEG-UAb), Aberta University, 1250-100 Lisboa, Portugal; 9 Nursing Research, Innovation and Development Center of Lisbon (CIDNUR), 1600-190 Lisboa, Portugal; 10 Instituto Superior de Engenharia de Lisboa (ISEL), Instituto Politécnico de Lisboa, 1959-007 Lisboa, Portugal; 11 Maxillofacial Surgery Unit, Department of Neurosciences, Reproductive and Odontostomatological Sciences, University of Naples “Federico II”, Via Sergio Pansini 5, 80131 Nápoles, Italy

**Keywords:** temporomandibular disorders, limited mouth opening, gender, diagnostic thresholds, personalized medicine

## Abstract

**Background/Objectives:** Temporomandibular disorder (TMDs) patients often present limited mouth opening (LMO). A key diagnostic cutoff is a mouth opening threshold >40 mm. However, this universal cutoff may not accurately reflect gender anatomical variations. This study investigates gender-specific differences in maximum mouth opening (MMO) to propose revised diagnostic criteria for LMO. **Methods:** A five-year prospective study was conducted from 1 August 2019 to 1 May 2024 in a Portuguese TMDs department. The patients’ gender, MMO, and LMO complaints with clinical validation were recorded. Statistical analyses, including Generalized Additive Models (GAMs) and Generalized Linear Models (GLMs), assessed the relationship between MMO and LMO, with gender-stratified comparisons. **Results:** In this study 1045 patients were included. The median (accompanied by the interquartile range [25th percentile–75th percentile]) MMO was lower in females (40 mm [34–45]) than in males (44 mm [40–48]). Patients presenting LMO complaints exhibited significantly reduced MMO values compared to those without LMO complaints (*p* < 0.001). Gender-specific thresholds emerged: for females, LMO was observed when MMO was ≤35 mm, while in males, LMO symptoms appeared when MMO was ≤38 mm. A “gray zone” of diagnostic uncertainty was identified between 36 and 37 mm for females and 38 and 42 mm for males. **Conclusions:** In this study we observed the gold standard cutoff for diagnosing MMO in female should be <35mm and for male <38mm. These findings suggest that a single LMO threshold does not account for gender-related anatomical differences, potentially leading to underdiagnosis in females and misclassification in males. Revising diagnostic criteria to incorporate gender-specific thresholds could enhance accuracy, improve early diagnosis, and promote personalized treatment strategies for TMDs patients. Further research incorporating additional variables such as age, dental occlusion, craniofacial structure, and body mass index is recommended to refine these diagnostic guidelines.

## 1. Introduction

Temporomandibular disorders (TMDs) are a group of conditions affecting the temporomandibular joint (TMJ), masticatory muscles, and associated structures, often resulting in pain, joint noises, and limited mouth opening (LMO). TMDs can significantly impact an individual’s quality of life by interfering with basic functions like chewing, speaking, and oral hygiene maintenance [1]. The estimated global prevalence of TMDs is approximately 34%, based on a recent meta-analysis, with regional variation across populations [2]. In Europe, the overall prevalence is 29%, with age-specific rates of 18% in individuals under 18 years old, 41% in adults aged 18–60, and 32% in individuals over 60 years old [2]. These figures underscore the widespread nature of TMDs and their clinical relevance across the lifespan.

LMO is one of the primary diagnostic criteria for TMDs, and it is typically assessed through a measurement of the interincisal distance during active mouth opening. The Diagnostic Criteria for Temporomandibular Disorders (DC/TMD) define LMO as an interincisal distance of less than 40 mm as the “cutoff” [3,4]. Despite the robustness of this threshold, it was established based on a study of over 20,000 individuals, which was conducted exclusively on healthy children aged 4–17 years. Other authors have suggested a cutoff of 35 mm [5,6]. These discrepancies indicate that relying on a fixed cutoff may lead to under- or overdiagnosis in certain subpopulations, emphasizing the need for personalized diagnostic criteria.

An emerging area of interest in the field of TMDs is the role of kinesiophobia, or fear of movement, in limiting mouth opening. Recent studies suggest that individuals with TMDs may reduce their range of jaw movement not only due to pain but also due to anticipatory fear of exacerbating symptoms, which contributes to a self-limiting behavioral pattern [7,8]. In this context, maximum mouth opening (MMO) is not solely a biomechanical measure but also a reflection of the patient’s psychological state. This fear-avoidance model has been linked to other musculoskeletal pain conditions and may have important implications for the interpretation of LMO in TMDs diagnosis.

Another critical dimension to consider is the influence of gender on MMO values. Numerous anatomical studies have documented significant gender-based differences in craniofacial morphology and muscle strength, with men typically exhibiting greater MMO due to larger jaw structures and more robust masticatory musculature [9,10,11,12]. Physiological factors such as hormonal differences and the increased prevalence of bruxism in women may also contribute to variations in MMO [13,14]. Bruxism, although not classified as a TMDs, is more prevalent among women and may contribute to increased muscle tension, fatigue, and joint overload [14,15]. This parafunctional activity can indirectly exacerbate or amplify TMDs-related symptoms, particularly in individuals with predisposing anatomical or psychological factors [16,17]. Psychological differences, including pain sensitivity, coping strategies, and the prevalence of anxiety-related disorders, may further modulate how TMDs presents and is perceived between genders [18,19].

Despite this well-established fact, the current 40 mm threshold is uniformly applied across genders. This uniform approach raises several concerns. Women, who naturally tend to have smaller MMO, may be underdiagnosed, for clinically significant limitations in mouth opening, leading to delays in treatment. Conversely, men may be overdiagnosed if their baseline MMO substantially exceeds the diagnostic threshold, resulting in unnecessary interventions or misclassification. The implications for clinical practice are substantial, suggesting that a one-size-fits-all model may not accurately identify and manage TMDs-related functional impairments.

This study argues that a single LMO threshold cannot capture gender differences in normal and pathological jaw function. The aim of this study is to analyze interincisal distances in male and female TMDs patients from a single-center clinical database, to evaluate whether gender-specific diagnostic thresholds for LMO are warranted. We hypothesize that gender differences in MMO are significant enough to justify the use of sex-specific thresholds for diagnosing LMO. Alternatively, if no significant gender-related differences are observed, this would support the continued use of a universal diagnostic threshold.

## 2. Materials and Methods

### 2.1. Study Design and Data Collection

**Figure 1:**
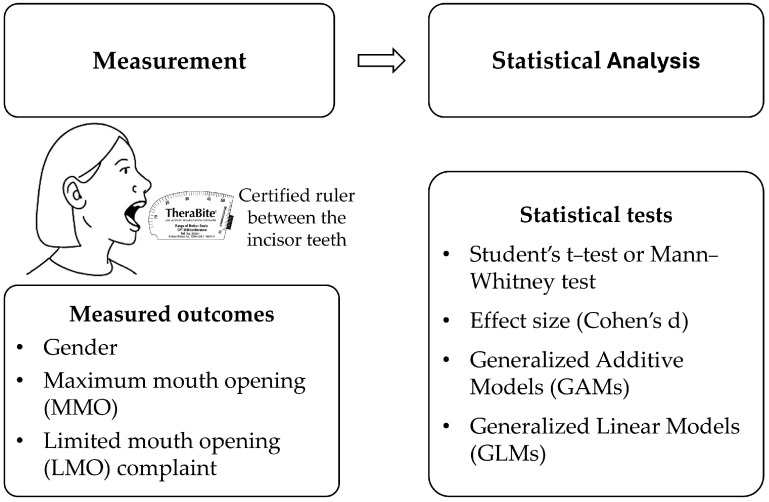
Flowchart illustrating the measurement of maximum mouth opening (MMO) using a certified ruler, and the main outcomes and statistical analyses included in this study.

A 5-year prospective study was conducted in a Portuguese TMDs department from August 1, 2019 to May 1, 2024. The Instituto Português da Face ethics committee approved this study (PT/IPFace/RCT/0525/19), and all of the enrolled patients provided their written informed consent in accordance with the current legislation. The inclusion criteria were as follows: (1) age ≥ 18 years; (2) recorded gender and MMO and LMO complaint during the first TMDs consultation; and (3) capacity to provide informed consent and participate in the clinical interview and examination (Figure 1). The exclusion criteria were as follows: (1) other facial problems that could potentially cause LMO (e.g., irradiation, cancer, trauma); (2) neuromuscular disorders or systemic conditions affecting mandibular function (e.g., Parkinson’s disease, multiple sclerosis); (3) severe psychiatric illness impairing communication or cooperation; and (4) any dental or prosthetic condition impeding an accurate MMO measurement. All eligible patients with inclusion criteria over these five years were included. Descriptive data and clinical outcomes were registered in the EUROTMJ database (https://eurotmj.org/tmj, accessed on 1 May 2025). At the first consultation, patients were instructed to answer questions regarding their complaints, namely LMO. To assess the presence of LMO, patients were asked a direct question during the initial consultation: “Do you feel that you have limited mouth opening?”. Those who responded affirmatively were further evaluated through a standardized clinical examination by the same experienced clinician (D.F.A) [4]. In clinical evaluation, the patients were instructed to perform a series of mouth-opening movements to observe jaw movement. LMO status was confirmed when the clinician verified a restricted mandibular range of motion consistent with the patient’s complaint. MMO (mm) was also registered. MMO was accessed using a certified ruler between the incisor teeth (TheraBite ® Jaw ROM Scale). All clinical evaluations were performed by the same clinician to ensure consistency (D.F.A).

### 2.2. Statistical Analysis

Analyses were conducted using the R Statistical language (version 4.3.3; R Core Team), on macOS (Apple M1 chip, 8-core CPU, 2020), using the packages likert (version 1.3.5; [20]), dplyr (version 1.1.4; [21]), reshape2 (version 1.4.4; [22]), ggstats (version 0.9.0; [23]), ggplot2 (version 3.5.2; [24]), GGally (version 2.2.1; [25]), caret (version 7.0-1 [26]), car (version 3.1-3; [27]), rpart (version 4.1.23; [28]), randomForest (version 4.7-1.2; [29]), pROC (version 1.18.5; [30]), pastecs (version 1.4.2; [31]), and rpart.plot (version 3.1.2; [32,33]). An exploratory analysis was conducted on the following variables by gender: LMO (–): absence of limitation or LMO (+): presence of limitation, and MMO. For these variables, appropriate measures of central tendency and dispersion, as well as frequency (both absolute and relative), were determined by gender. The sample mean accompanied by the standard deviation was used for approximately symmetric distributions, presented as the mean (standard deviation). For asymmetric distributions, the median accompanied by the interquartile range was reported, in the format of the median [P25; P75]. Skewness was assessed through a hypothesis test on the population skewness coefficient.

The normality of MMO distribution was assessed using the Shapiro–Wilk test. Homoscedasticity was tested using Bartlett’s test upon confirming normality in the MMO distribution across LMO categories. If both conditions were met, Student’s parametric t-test was applied. In cases where homoscedasticity was violated, Welch’s parametric test was used. The absence of normality led to the choice of the non-parametric Mann–Whitney test.

Effect size was measured using Cohen’s d to compare MMO between female and male participants, between LMO (–) and LMO (+), and within each gender group (female and male). Cohen’s d quantifies the magnitude of the difference between groups in terms of the pooled standard deviation. A larger d value indicates a greater difference, with conventional thresholds for interpretation: small (0.1), medium (0.3), and large (0.7) [34]. This approach helps to assess the practical significance of differences in MMO both between genders and within each gender group based on LMO status.

After completing the exploratory analysis, a study of the functional relationship between MMO and LMO by gender was conducted using Generalized Additive Models (GAMs). In this class of models, the logistic link function was used due to the response variable (LMO) following a Bernoulli distribution. Where the functional form appeared to be approximately linear, the association was modeled using Generalized Linear Models (GLMs) with the same link function as in the GAMs. From the GLMs, the respective odds ratios of MMO by gender were estimated. An alpha significance level of 5% was set.

## 3. Results

A total of 1045 patients were analyzed using records from the EUROTMJ database. The exploratory analysis results are summarized in Table 1. In this table, the MMO measures account for the fact that the distributions by gender are asymmetrical (female 80%; male 20%).

**Figure 2:**
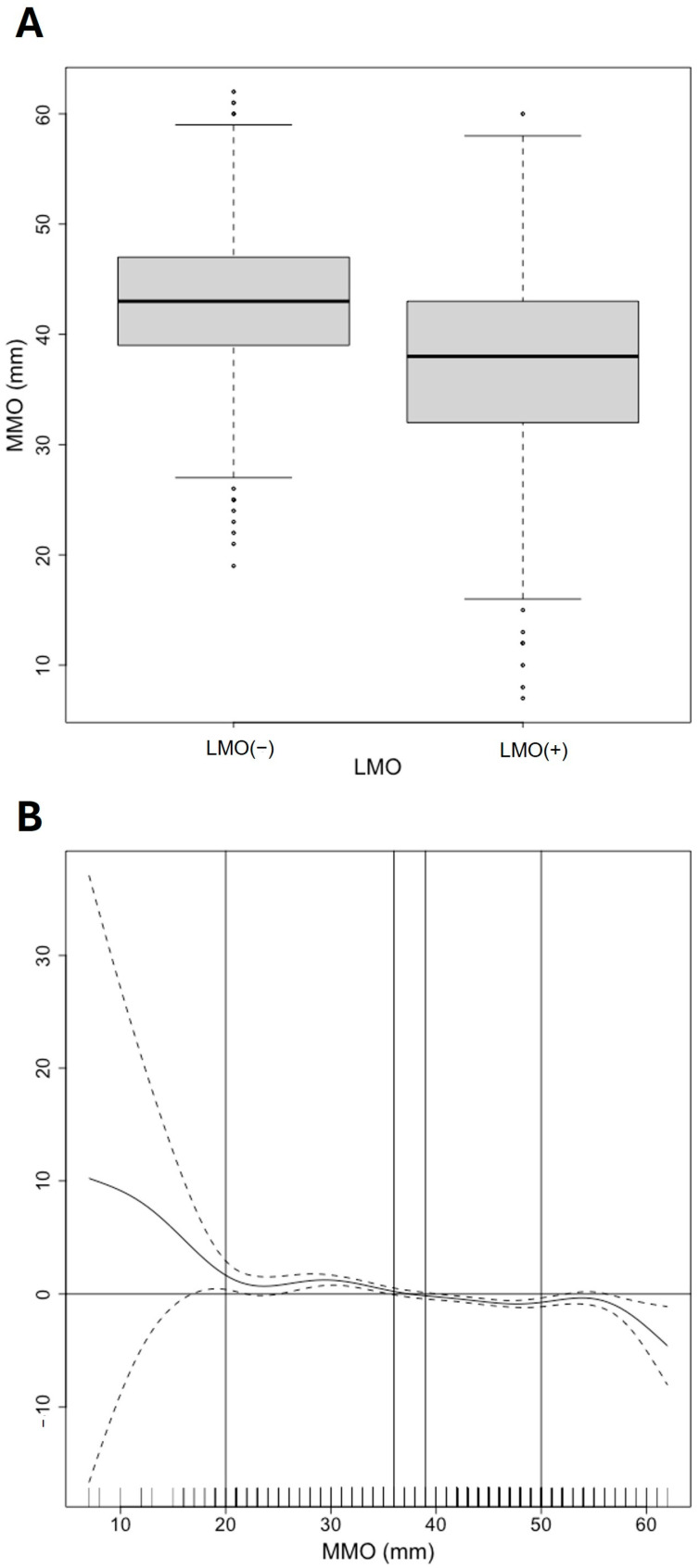
Maximum mouth opening (MMO) values among patients with limited mouth opening (LMO) complaints. (**A**) The boxplot represents the distribution of MMO between each category of LMO. (**B**) Generalized Additive Model (GAM) for describing the functional form of the association between the MMO and LMO variables. For statistical analysis, only the MMO values limited by the vertical lines 20 and 50 mm, respectively, were considered given the low number of cases outside this range. The risk of LMO (+) for MMO values between the 36 and 39 mm vertical lines, respectively, is undefined since the confidence bands include the zero value of the ordinate. Values below 36 mm correspond to a risk of LMO (+). Values greater than 39 mm correspond to LMO (–).

A comparison of MMO values between patients who were positively and negatively diagnosed with LMO was conducted. Figure 2A shows that the MMO medians are different. This evidence is supported by comparing the difference in median values (*p* < 0.001). The corresponding Cohen’s d value was 0.645, which indicates a medium effect size. The median MMO values in the LMO (–) group were 43.0 mm [39.0;47.0], and in the LMO (+) group, they were 38.0 mm [32.0;43.0]. Subsequently, a GAM was applied to describe the functional form of the association between the MMO and LMO. Due to the number of observations being lower outside the MMO interval [20;50] mm, we will consider only values belonging to it. Patients present a risk of LMO symptoms in cases where MMO < 36 mm. In cases where the MMO is >39 mm, the risk of not having LMO symptoms increases. Given the near linearity of the functional form, a GLM with a logistic link function was then applied to model the association between MMO and LMO. This model indicates that the MMO has statistical significance (*p* < 0.001) regarding the Wald test application. Additionally, the GLM provided a 95% confidence interval for the odds ratios [0.903; 0.935], indicating that for each additional 1 mm increase in MMO, the odds ratio of LMO decreases by 1.065; 1.097.

**Figure 3:**
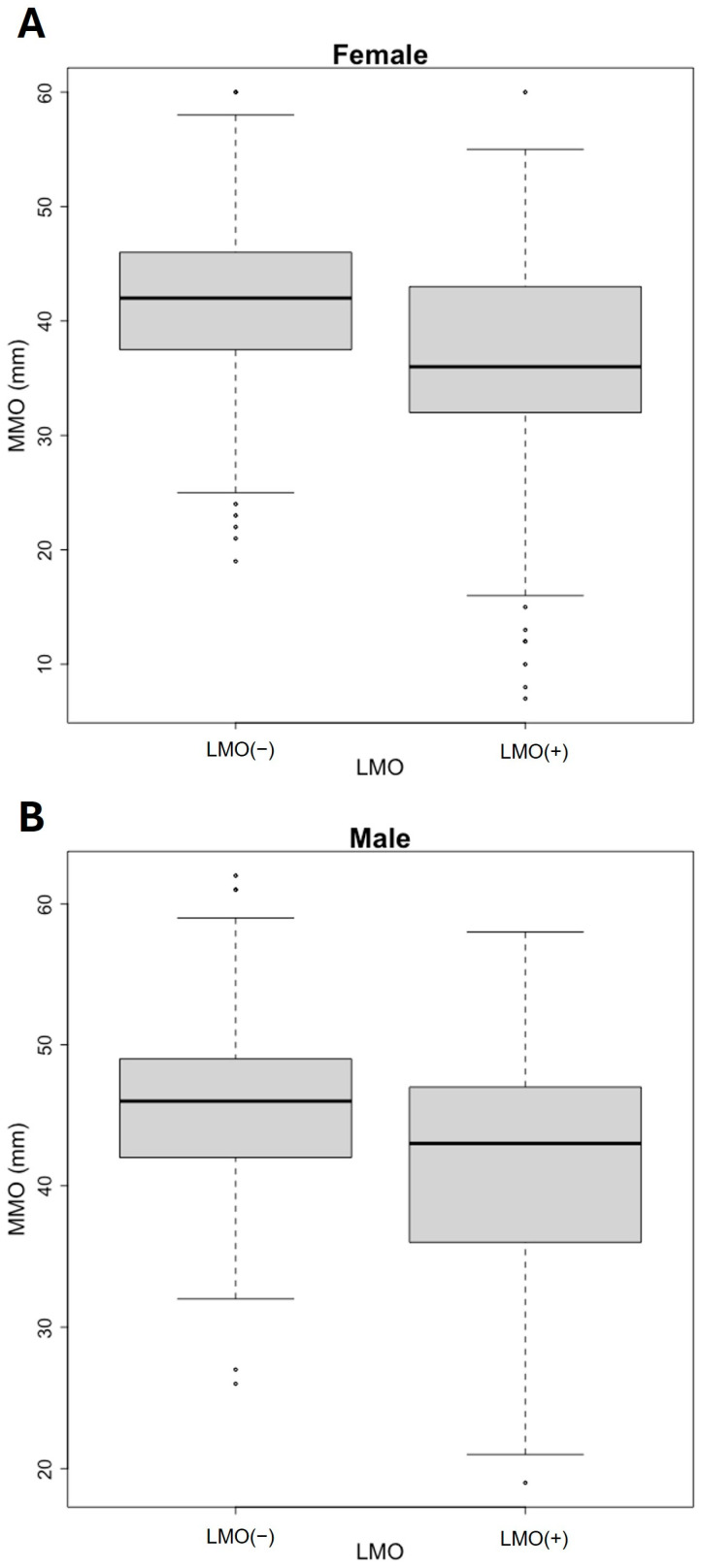
Maximum mouth opening (MMO) values among female (**A**) and male (**B**) patients with limited mouth opening (LMO (+) and without LMO (−)). The boxplot represents the distribution of MMO between each category of LMO.

Subsequently, a comparative analysis was conducted on MMO values regarding gender. The median MMO values were compared considering the presence or absence of LMO for females. It was found that the median of MMO in LMO (−) was 42.0 mm [37.5; 46.0], whereas in the LMO (+), the value was 36.0 mm [32.0;43.0] (Figure 3A). Statistical significance was observed when comparing MMO in the two groups (*p* < 0.001). The corresponding Cohen’s d value was 0.555, which indicates a medium effect size. For males, the median of MMO in the LMO (−) group was 46 mm [42;49], while in the LMO (+) group, the value was 43 mm [36;47] (Figure 3B). Statistical significance was also observed when comparing the two groups (*p* < 0.001). The corresponding Cohen’s d value was 0.623, which also indicates a medium effect size.

**Figure 4:**
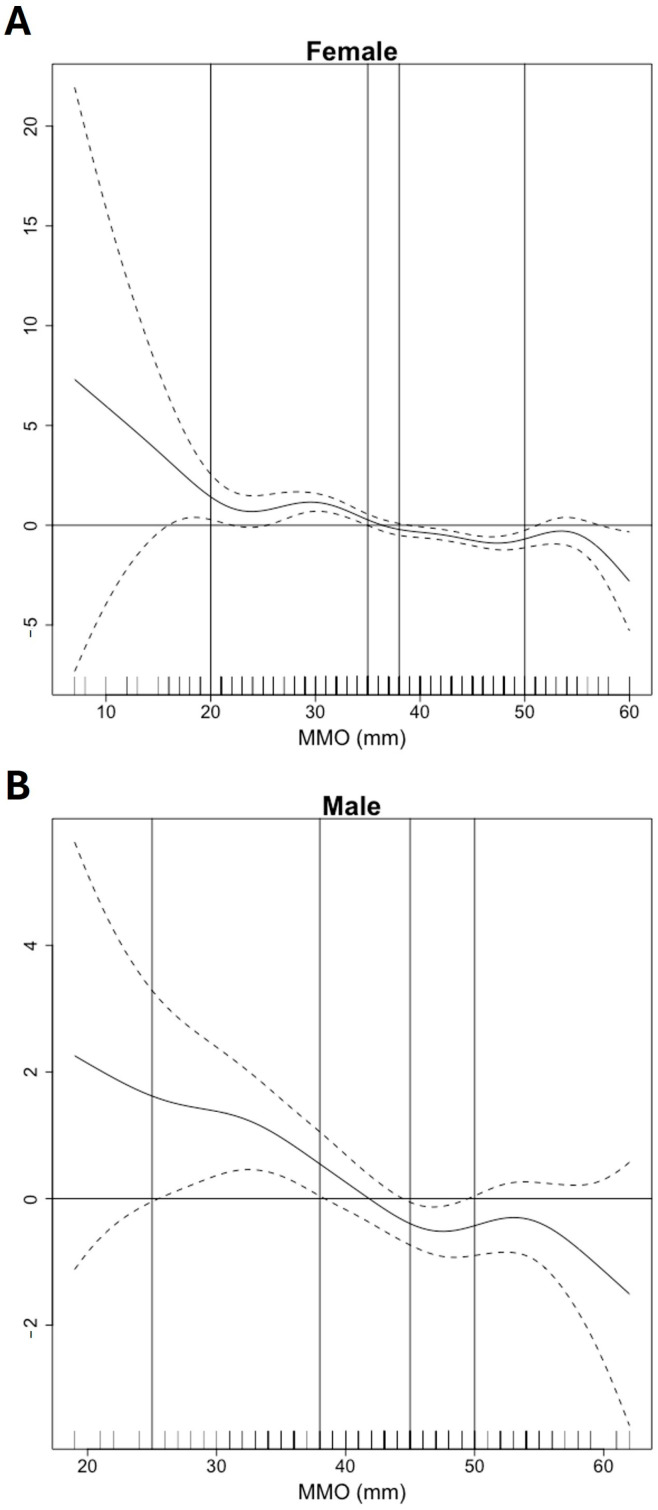
Functional distribution of maximum mouth opening (MMO) values among female (**A**) and male (**B**) patients with limited mouth opening (LMO (+) and without LMO (−)). Generalized Additive Model (GAM) for describing the functional form of the association between MMO and LMO variables. For statistical analysis, only the MMO values limited by the vertical lines 20 and 50 mm, respectively, were considered given the low number of cases outside this range. The risk of LMO (+) for MMO values between the 36 and 39 mm vertical lines, respectively, is undefined since the confidence bands include the zero value of the ordinate. Values below 35 and 38 mm correspond to a risk of LMO (+) for females and males, respectively. Values greater than 38 and 45 mm correspond to LMO (−) for females and males, respectively.

The GAM was then applied to describe the functional form of the association between the MMO and LMO for both genders (Figure 4A,B). For females, LMO (+) is present if the MMO is ≤35 mm. When the MMO is >38 mm, the risk of LMO decreases. The GLM provided a 95% confidence interval for the odds ratios [0.901; 0.938], indicating that for each additional 1 mm increase in MMO, the odds ratio of LMO (+) decreases by 1.061; 1.099. For males with LMO (+), the symptoms are present if the MMO is less than 38 mm. The risk of LMO decreases when the MMO is >45 mm. The GLM provided a 95% confidence interval for the odds ratios [0.889; 0.963], indicating that for each additional 1 mm increase in MMO, the risk of LMO (+) decreases by 1.037; 1.111. In a univariate logistic regression model using MMO as the sole predictor of LMO status, the area under the ROC curve (AUC) was 0.69 for both males and females. This indicates a moderate discriminatory ability, suggesting that MMO alone can partially distinguish between patients with and without perceived LMO.

## 4. Discussion

This study emphasizes the potential for gender-specific thresholds in diagnosing LMO associated with TMDs, as it uncovers significant differences in MMO capacities between males and females. By examining MMO variations in male and female TMDs patients using flexible statistical models, this article demonstrates consistent sex-based differences that question the validity of a universal diagnostic threshold. Specifically, females displayed significantly lower MMO values, justifying a diagnostic threshold of 35 mm, while for males, 38 mm was more appropriate. Diagnostic gray zones—ranging from 36 to 37 mm for women and 38 to 42 mm for men—further reflect natural inter-individual variation and emphasize the limitations of applying a single cutoff point across all patients.

Well-established differences in craniofacial morphology and biomechanics support the biological underpinnings of these findings [35]. Males generally possess larger mandibular structures, wider condylar heads, and longer ramus lengths, contributing to a greater mandibular excursion capacity and, consequently, higher MMO values [36,37]. Women, in contrast, exhibit more gracile facial bone structure, reduced muscle cross-sectional area, and different TMJ geometry, all of which may contribute to narrower physiological ranges for mouth opening [10,36,37,38]. Additionally, hormonal differences influence soft tissue behavior and pain perception. Estrogen, for example, is known to increase ligament laxity and may sensitize nociceptive pathways, potentially influencing both joint mobility and pain reporting in female patients [39,40]. Beyond anatomical factors, neurophysiological and psychosocial influences are increasingly recognized as contributors to sex differences in TMDs [41]. Women are more likely to experience chronic pain syndromes and often score higher on psychological distress measures, including anxiety, depression, and somatization, all of which are linked to increased TMDs severity and functional limitation [40]. Kinesiophobia, the fear of movement due to the anticipation of pain, may limit voluntary jaw opening and perpetuate dysfunction [7,8]. This behavioral pattern, more commonly observed in women, reinforces the argument for gender-sensitive diagnostic tools [7,8]. Recent studies have highlighted the significant impact of psycho-emotional factors on TMDs. For instance, a pilot study utilizing a specialized questionnaire found that TMDs patients exhibited higher levels of psychological distress compared to healthy controls, emphasizing the need for psychological counseling in TMDs management [42]. Recently, our investigation group showed significant correlations between clinical satisfaction and factors such as patient expectations and post-treatment pain levels. Notably, the presence of depression was associated with an increased need for further TMJ treatment, highlighting the importance of addressing psychological factors in TMDs management [18]. Additionally, stress has been shown to influence TMJ status and salivary cortisol levels, particularly during high-stress periods such as exams, suggesting a direct link between stress and TMDs symptoms [43]. Another key factor is the higher prevalence and severity of bruxism among women [15]. Although bruxism is not classified as a TMDs, bruxism can result in chronic hyperactivity of the masticatory muscles, leading to fatigue, pain, and reduced MMO [44,45]. Studies have shown that women who brux are more likely to develop myofascial pain and muscle guarding, which can further restrict jaw function [15,46]. In combination with higher pain sensitivity and greater stress reactivity, this suggests a feedback loop that disproportionately affects female patients and contributes to lower MMO measurements. This study reflects the clinical reality where bruxism often coexists with TMDs, particularly in patients with myofascial pain or functional limitations.

From a clinical standpoint, the debate over threshold values for diagnosing LMO has long been contentious. MMO is typically measured as the interincisal distance, which is the space between the upper and lower central incisors when the mouth is fully opened, and used to define LMO. The DC/TMD, established by the International RDC/TMD Consortium Network and the Orofacial Pain Special Interest Group, set the threshold for diagnosing LMO at an interincisal distance of less than 40 mm [3,4]. This value is derived as a clinical standard for identifying significant restrictions in mouth opening that may indicate TMDs, specifically in cases where disc displacement without reduction is present. According to the DC/TMD guidelines, if passive mouth opening (with assistance) remains below this 40 mm threshold, it strongly suggests the presence of LMO, making it a critical diagnostic marker for TMDs [4]. However, LMO should not be interpreted in isolation; its diagnostic value is enhanced when considered alongside other clinical findings such as joint pain, joint sounds, muscle sensitivity, and functional limitation. The support for using 40 mm in this criterion was based on a study of around 20,000 children/adolescents [3,4]. In contrast, other diagnostic systems, such as the Dijkstra classification for trismus, recognize a wider spectrum of functional limitation: mild (30–35 mm), moderate (15–30 mm), and severe (<15 mm) [6]. Mild trismus corresponds to a mouth opening of 30 to 35 mm, moderate trismus is defined by 15 to 30 mm, and severe trismus involves a mouth opening of less than 15 mm. Our findings align more closely with this graded classification and suggest that rigid dichotomous cutoffs may be clinically limiting, especially when applied without consideration of patient sex. The accuracy of MMO measurements is crucial for proper diagnosis. A recent study evaluated the reproducibility and validity of various MMO measurement techniques, highlighting the importance of standardized methods to ensure consistent and reliable assessments across different patient populations [47].

Methodologically, the use of GAMs and GLMs in this study provides strong statistical support for individualized threshold modeling. These models accommodate non-linear relationships and allow for the better estimation of probability curves across MMO values. The finding that each millimeter increase in MMO is inversely associated with the likelihood of LMO—and that this relationship is stronger when stratified by sex—supports the need for personalized diagnostic criteria. Such individualized cutoffs may help clinicians identify early-stage TMDs in male patients whose MMO remains above 40 mm, and more accurately diagnose LMO in women whose MMO, while below 40 mm, may still be within their normal range. The analysis revealed an area under the curve (AUC) of 0.69 for both males and females, indicating moderate discriminatory power. This suggests that MMO alone has some ability to distinguish between patients with and without LMO, performing better than random chance but falling short of high diagnostic accuracy. These findings reinforce the idea that MMO, while informative, may be more valuable as part of a multivariable diagnostic model that incorporates additional anatomical, functional, or psychosocial variables. The moderate AUC also supports our rationale for not relying on a single fixed threshold for diagnostic classification but instead exploring flexible, sex-specific cutoffs tailored to individual patient characteristics.

The implications of these findings extend across diagnostic, rehabilitative, and educational domains. Clinically, applying gender-specific thresholds could significantly reduce underdiagnosis in women and prevent overdiagnosis in men, enhancing diagnostic accuracy. The more accurate identification of LMO can also inform tailored rehabilitation plans, including physiotherapy, occlusal therapy, and behavioral interventions such as biofeedback or cognitive behavioral therapy (CBT) for kinesiophobia. Furthermore, patient education can be improved when normative values are presented within a personalized, gender-informed framework, fostering trust and engagement in the therapeutic process. Moreover, this approach aligns with the principles of personalized medicine, which prioritize individualized assessment and treatment planning over population-based generalizations. Future clinical guidelines for TMDs assessment may benefit from incorporating sex-specific diagnostic algorithms, especially in multidisciplinary settings involving dentists, physiotherapists, and orofacial surgeons.

This study does have limitations that impact the generalizability of its findings. Notably, the cross-sectional design restricts the ability to track the progression of MMO and LMO in TMDs patients over time, which would provide more comprehensive insight into the role gender plays in TMDs onset and prognosis. One of the limitations of this study is the inclusion of patients with various types of TMDs, including both arthrogenous (e.g., disc displacement, osteoarthrosis) and myogenous (e.g., myofascial pain) conditions. These subtypes may affect MMO through different pathophysiological pathways—structural restriction versus muscular guarding—potentially introducing clinical heterogeneity into the dataset. While this reflects the real-world diversity of TMDs presentations, future studies should consider stratifying participants by diagnostic category to allow for more specific and reliable conclusions regarding sex-based diagnostic thresholds. Another limitation is that this study included patients with self-reported or clinically suspected bruxism, which, although not classified as a TMDs, may influence masticatory muscle activity and confound the assessment of MMO. While this reflects real-world clinical presentations, it may limit the specificity of findings related solely to TMDs pathophysiology. Additionally, while this study’s sample size is robust, confounding variables like age, craniofacial structure, dental occlusion, BMI, and ethnicity—which can significantly affect MMO—were not controlled. Although age was recorded, it was not incorporated into the statistical models as a covariate. These variables may influence baseline mandibular mobility and the clinical response to intra-articular interventions, particularly in relation to MMO. Their omission limits the ability to perform stratified analyses or identify subgroups with differential treatment responses. A longitudinal study approach, incorporating these factors, could offer a more nuanced understanding of MMO variations and refine gender-specific thresholds for broader populations.

Although our study did not directly evaluate the influence of age, BMI, occlusal status, or craniofacial structure on maximum mouth opening (MMO) outcomes, these factors are known to influence mandibular kinematics and are functional limitations in patients with TMDs. Age-related changes in joint elasticity, muscle tone, and intra-articular degeneration may restrict the mandibular range of motion and affect responsiveness to treatment [48]. Similarly, elevated BMI has been associated with systemic inflammation and increased mechanical load on joints, potentially limiting mandibular excursion [48,49]. Occlusal discrepancies and craniofacial morphology, including Class II or III skeletal patterns, deep bite, or mandibular retrognathism, have been shown to alter the biomechanics of the TMJ and may predispose patients to hypomobility or disc interference [50,51,52]. Stratifying analyses or incorporating these variables into multivariable or interaction-based models could provide a more refined understanding of patient-specific risk factors for LMO and improve the accuracy of individualized diagnostic thresholds. These variables were not fully explored in the current analysis; their potential confounding or moderating roles merit consideration in future studies. From a statistical standpoint, future studies should consider incorporating these patient-specific characteristics as covariates or interaction terms in linear mixed-effects models or ANCOVA designs. This would allow for the more refined analysis of treatment effects on MMO, helping to determine whether specific subgroups (e.g., younger patients, normo-weight individuals, or those with balanced occlusion) respond more favorably to HA+PRP interventions. Stratified analysis could also support the development of more personalized treatment algorithms in TMJ rehabilitation.

## 5. Conclusions

In conclusion, this study provides compelling evidence for revisiting and refining the diagnostic criteria for TMDs through a gender-specific lens. Identifying distinct thresholds for LMO in men and women highlights the limitations of a universal diagnostic cutoff and underscores the value of incorporating anatomical, physiological, and behavioral differences into clinical assessment protocols. In this study we observed the gold standard cutoff for diagnosing MMO in female should be <35mm and for male <38mm.

While these findings are promising, they also reveal the need for more comprehensive research to validate and expand upon this work. Future studies should adopt longitudinal, multicenter designs to evaluate changes in MMO over time and across treatment stages. Tracking hormonal fluctuations, especially in relation to menstrual cycle phases and menopausal status, may provide deeper insights into the role of sex hormones in TMDs symptom expression and MMO variability. Incorporating psychometric assessments—such as measures of anxiety, depression, and kinesiophobia—can further elucidate the interplay between psychological stress and functional limitation in TMDs patients. Additionally, machine learning techniques hold potential for developing predictive models that integrate demographic, anatomical, psychological, and behavioral data to establish personalized diagnostic thresholds.

By advancing toward individualized diagnostic criteria, healthcare professionals can improve diagnostic accuracy, avoid misclassification, and deliver more effective, targeted interventions. These refinements enhance clinical outcomes and align with broader healthcare trends prioritizing precision and patient-centered care.

Applying gender-specific thresholds for LMO can improve diagnostic accuracy, reduce misclassification, and lead to more tailored treatment planning for TMDs patients. Recognizing that men and women differ in their physiological mandibular capacity allows clinicians to avoid underdiagnosis in females and overtreatment in males. These findings can directly inform clinical protocols for diagnosis, rehabilitation, and patient education, contributing to more precise and individualized care in orofacial pain management.

## Figures and Tables

**Table 1:** Demographic data by gender according to maximum mouth opening (MMO) and limited mouth opening (LMO).

Variables	N (%) or Median [P_25_;P_75_]	
Number of patients		1045
Gender	Female	836 (80%)
	Male	209 (20%)
MMO (mm)		41.0 [34.0;45.0]
	Female	40.0 [34.0;45.0]
	Male	44.0 [40.0;48.0]
LMO (+)		633 (60.6%)
	Female	529 (63.3%)
	Male	104 (49.8%)
LMO (–)		412 (39.4%)
	Female	307 (36.7%)
	Male	105 (50.2%)
MMO (mm)		
	LMO (–)	43.0 [39.0;47.0]
	LMO (+)	38.0 [32.0;43.0]

## Data Availability

Data are contained within the article.

## Abbreviations

AUC: area under the curve; BMI: Body Mass Index; CBT: cognitive behavioral therapy; DC/TMD: Diagnostic Criteria for Temporomandibular Disorders; GAM: Generalized Additive Model; GLM: Generalized Linear Model; IQR: interquartile range; LMO: limited mouth opening; MMO: maximum mouth opening; ROC: receiver operating characteristic; TMDs: temporomandibular disorders; TMJ: temporomandibular joint.
